# Transcriptional and reverse transcriptional regulation of host genes by human endogenous retroviruses in cancers

**DOI:** 10.3389/fmicb.2022.946296

**Published:** 2022-07-19

**Authors:** Mengwen Zhang, Shu Zheng, Jessie Qiaoyi Liang

**Affiliations:** ^1^The Second Affiliated Hospital of Zhejiang University School of Medicine, Hangzhou, China; ^2^Ministry of Education Key Laboratory of Cancer Prevention and Intervention, Second Affiliated Hospital, Cancer Institute, Zhejiang University School of Medicine, Hangzhou, China; ^3^Department of Medicine and Therapeutics, Faculty of Medicine, Center for Gut Microbiota Research, Li Ka Shing Institute of Health Sciences, Shenzhen Research Institute, The Chinese University of Hong Kong, Hong Kong, Hong Kong SAR, China

**Keywords:** endogenous retrovirus (ERV), long terminal repeat (LTR), cancer, non-coding RNA, reverse transcription, retrocopying

## Abstract

Human endogenous retroviruses (HERVs) originated from ancient retroviral infections of germline cells millions of years ago and have evolved as part of the host genome. HERVs not only retain the capacity as retroelements but also regulate host genes. The expansion of HERVs involves transcription by RNA polymerase II, reverse transcription, and re-integration into the host genome. Fast progress in deep sequencing and functional analysis has revealed the importance of domesticated copies of HERVs, including their regulatory sequences, transcripts, and proteins in normal cells. However, evidence also suggests the involvement of HERVs in the development and progression of many types of cancer. Here we summarize the current state of knowledge about the expression of HERVs, transcriptional regulation of host genes by HERVs, and the functions of HERVs in reverse transcription and gene editing with their reverse transcriptase.

## Introduction

Human endogenous retroviruses (HERVs) are the remnants of infectious retroviral agents, which were initially integrated into the genome of human germline cells for vertical genetic transmission. HERVs have accumulated in the human genome for over 60 million years since initial germline fixation, expanding as retrotransposons, and have been inherited through successive human generations in a Mendelian fashion (Jaenisch, 1976; Feschotte and Gilbert, 2012). HERVs account for ∼8% of our genome (Lander et al., 2001). The identification and phylogenetic analysis of HERVs were accelerated by the use of Whole-genome sequencing technology. Generally, HERVs are classified by their relationship with exogenous retroviruses and named by the specific tRNAs of their primer binding sites (PBSs) that are involved in their reverse transcription. Fast progress in deep sequencing and functional analysis has revealed the importance of domesticated copies of transposable elements (TEs), including their regulatory sequences, transcripts, and proteins in normal cells. HERVs are highly active in embryonic and pluripotent cells but mostly remain silenced in differentiated cells (Grow et al., 2015). Recently, evidence suggests that HERVs are involved in the development and progression of various cancer (Staege and Emmer, 2018; Zhang et al., 2019). As ERV sequences are resident in all human cells whole life long, they were likely to have considerable impacts on the human genome and biological activity, while they are not fully understood yet. We summarize the expression of HERVs in normal cells and cancers, their transcriptional regulation of host genes, and the functions of HERVs in RT and gene editing with their reverse transcriptase (RTase).

## Human endogenous retroviruses and their physiological expression in the human genome

### Human endogenous retroviruses composition in the human genome

The human genome contains all the information about human development, variation, and evolution. Marked variation of different features was shown by the genomic landscape, including genes, TEs, recombination, and so on. The human genome project revealed that retrotransposable elements account for about 45% of the human genome, while the exons of our protein-coding genes represent only 1.2%, and dozens of genes seem to be derived from TEs (Redi and Capanna, 2012). TEs were composed of retrotransposons (class 1) and DNA transposons (class 2). Retrotransposons are subdivided by whether there are long terminal repeats (LTRs) flanking the central coding region. Non-LTR retrotransposons are divided into “autonomous” and “non-autonomous” elements, depending on whether they can retrotransposition within themselves (Platt et al., 2018). The most abundant classes of autonomous retrotransposons and endogenous RTase elements are the long interspersed nuclear elements-1 (*LINE-1*) and *Alu* (Lander et al., 2001), while ERVs are retrotransposons with LTRs flanking coding region.

ERV sequences were highly similar to exogenous retroviral proviruses as they have originated by RT into DNA from infectious retroviruses and then integrated into the host genome long ago. An ERV genome generally consists of four genes encoding different proteins and enzymes: *gag*, *pro*, *pol*, and *env*. *Gag* encodes structural matrix and capsid proteins; *pro* encodes the viral protease; *pol* encodes the RTase and integrase; and *env* encodes envelope glycoproteins that mediate the host cell tropism. ERV sequences were highly variable, while they also preserved some features of the original provirus. Some ERV sequences preserve complete LTRs but not the retroviral genes, most of which are highly fragmented as compared with their parental virus genomes, losing most of their coding capacities. Retroviral genes *Syncytin-1* (Mboko et al., 2014), *Rec*, and *Np9* (Uygur et al., 2019) still retain the protein-coding capacity, encoding fully functional proteins. These retroviral genes belong to HERVs (HERV-W and HERV-K) that have been incorporated into the genome relatively recently. However, there seems to be no fully infectious HERVs anymore.

### Expression of human endogenous retroviruses in normal cells

HERVs exist in all human cells, but their expression differs greatly at mRNA and protein levels (Lower et al., 1993). Most HERVs are silent in healthy adult cells and tissues, but proviral RNAs or proteins were observed in several diseases, such as inflammation, autoimmune diseases, and malignancies (Grandi and Tramontano, 2018a; Tokuyama et al., 2018; Licastro and Porcellini, 2021). Recently, several studies reported that *HERV-K* expression was increased after severe acute respiratory syndrome coronavirus 2 (SARS-CoV-2) infection both *in vivo* and *in vitro* (Guo et al., 2022; Temerozo et al., 2022). Interestingly, evidence showed that HERV activity correlated with cell proliferation. HERVs were highly active in early embryonic cells and in induced pluripotent stem cells (iPSC), where they seem to be indispensable but still need tightly regulated for successful differentiation (Grow et al., 2015). HERVs were demonstrated to be highly expressed in reproductive organs and embryonic origin, while mature, terminally differentiated and non-dividing muscle cells showed less HERV activity (Seifarth et al., 2005). Expression profiles of several proviruses of the HERV-K and HERV-W families show an aging-related pattern as indicated by genome-wide RNA-sequencing (Nevalainen et al., 2018). Tokuyama et al. (2018) described a method called ERV map to study cell-type-specific ERV expression patterns in specific cells or tumor tissues. ERV map across various diseases has the potential to discover new disease-associated antigens that have not been identified currently by focusing on protein-coding sequences.

## Human endogenous retroviruses in carcinogenesis

### Aberrant reactivation of human endogenous retroviruses in cancers

**FIGURE 1:**
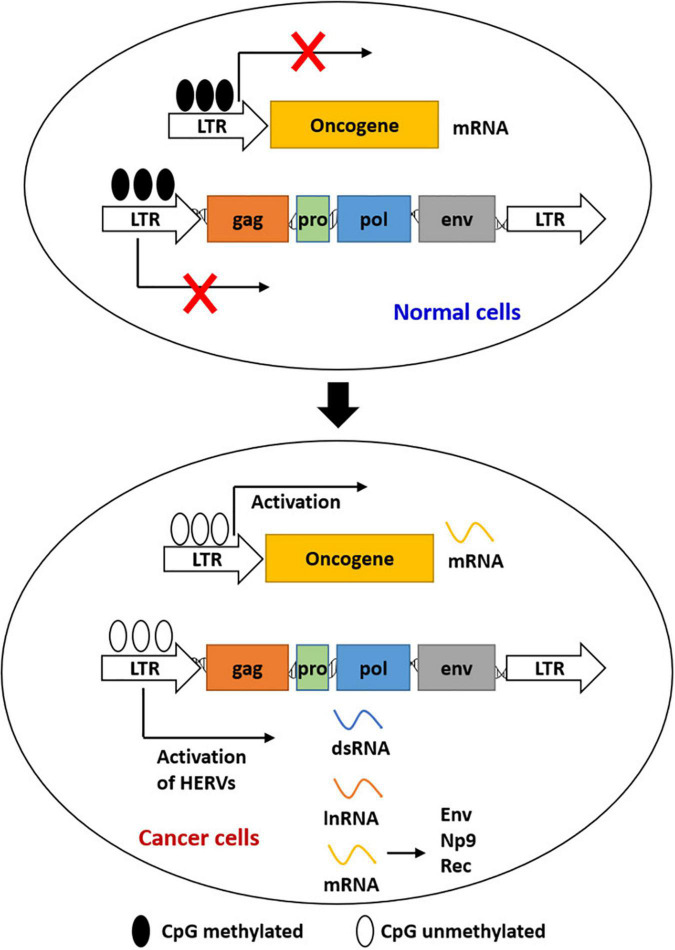
Epigenetic regulation of the expression of HERVs and LTR-driven genes during carcinogenesis. Most HERVs/LTRs are inactivated in normal cells by DNA methylation. Activation of HERVs is induced by the global loss of DNA methylation in cancer cells. Activated HERVs can be transcribed into mRNAs, lncRNAs, and dsRNAs. LTRs can also act as the promoters of host genes to induce gene expression.

Cancer remains a major challenging disease globally. Viral products of HERVs were demonstrated to have played a role in species evolution, as well as cancer development (Ruprecht et al., 2008; Staege and Emmer, 2018). The best-known mechanisms for HERV expression and repression were epigenetic mechanisms (Figure 1), such as DNA methylation and histone modifications (Xie et al., 2013). Epigenetic alterations were reported to be responsible for the initiation of tumor development to some extent, including hypermethylation of tumor suppressor genes and hypomethylation of oncogenes (Rodriguez-Paredes and Esteller, 2011). Retroviral elements that promote carcinogenesis were expressed again by reactivation of HERVs, which was regulated by epigenetic reprogramming (Staege and Emmer, 2018; Zhang et al., 2019). HERV RNAs and HERV-encoded proteins were detected in various cancers including germ cell tumors, melanoma, genital tract cancers, gastrointestinal cancer, and breast cancer (Table 1).

**TABLE 1:** Expression of HERV families in cancers.

HERV family	Cancer type
HERV-E	Clear cell kidney cancer (Cherkasova et al., 2011), breast cancer (Frank et al., 2008)
HERV-F	Soft tissue sarcoma (Giebler et al., 2018), breast cancer (Frank et al., 2008)
HERV-H	Gastrointestinal and pancreatic neuroendocrine tumors (Yuan et al., 2021), colon cancer (Liang et al., 2009a,b, 2012; Li et al., 2012), lung cancer (Zare et al., 2018), breast cancer (Rhyu et al., 2014)
HERV-K	Breast cancer (Wang-Johanning et al., 2003), soft tissue sarcoma (Giebler et al., 2018), melanoma (Singh et al., 2013, 2020), germ cell tumor (Chan et al., 2019), leukemia (Chen et al., 2013), colon cancer (Dolci et al., 2020a,b), Hodgkin lymphoma (Barth et al., 2019), lung cancer (Zare et al., 2018)
HERV-P	Lung cancer (Zare et al., 2018), breast cancer (Tavakolian et al., 2019), primary cutaneous t-cell lymphomas (Bergallo et al., 2018)
HERV-R	Colon cancer (Dolci et al., 2020a), lung cancer (Zare et al., 2018), breast cancer (Rhyu et al., 2014), ovarian cancer (Jeon et al., 2020), primary cutaneous T-cell lymphomas (Bergallo et al., 2018)
HERV-W	Endometrial (Strissel et al., 2012), testicular cancer (Gimenez et al., 2010), germ cell tumors (Benesova et al., 2017), non-small cell lung cancer (NSCLC) (Li et al., 2019), endometrial carcinoma (Liu et al., 2019)
HERV-FRD	Seminomas (Benesova et al., 2017), glioblastoma (Diaz-Carballo et al., 2017)
HEMO	breast cancer and ovarian cancer (Heidmann et al., 2017), endometrial cancer (Kasperek et al., 2021)

Since the expression of HERVs is associated with cancers to some extent, HERVs may act as biomarkers for cancers. Song et al. (2021) have reviewed the HERVs as biomedicine markers and provided a new perspective on the clinical application of HERVs. The newly discovered “HEMO” HERV envelope (Env) protein, encoded by the MER34 member 1 of the ERV group, was active in numerous cancerous tissues and most active in breast cancer and ovarian cancer (Heidmann et al., 2017). Recently, Kasperek et al. (2021) reported a pan-cancer analysis of HEMO both in primary tumors and metastatic tumors. A link between HEMO expression and Wnt/β-catenin signaling activation was revealed in endometrial cancer (Kasperek et al., 2021). The aberrant reactivation of HERV-H in cancers, especially colorectal cancer, has been summarized in our previous review (Zhang et al., 2019). Dynamic roles of the aberrantly activated HERVs have been reported in cancer development. HERVs have also been reported to be a promising predictive biomarker for anti-cancer therapy. Ficial et al. (2021) revealed that high levels of ERVE-4 expression combined with CD8^+^PD1^+^TIM3^–^LAG3^–^ tumor-infiltrating cells predicted response to the anti-PD-1 antibody nivolumab in patients with metastatic clear cell renal cell carcinoma.

### Human endogenous retroviruses-mediated mechanisms of oncogenesis

HERVs play oncogenic roles *via* various mechanisms, including transforming properties of the HERV genes, direct participation in the maintenance of cancer phenotypes, inactivation of tumor suppressor genes, activation of oncogenes, mediating cell fusion, activation of cancer signaling pathways, suppression of anti-tumor immunity, etc.

Some retroviruses are proven to have transforming properties *in vivo*, characterized by the presence of host oncogenes in their genomes during retroviral transduction (van Lohuizen and Berns, 1990). Some transforming retroviruses carry oncogenes themselves, such as those with the oncogene *ra*s named after rat sarcoma, which is homologous to human oncogenes named *HRAS, KRAS*, and *NRAS* (Weiss, 2020). The functional proteins encoded by HERVs also have oncogenic or transforming potentials. Recent research showed the ERK1/2 pathway was activated by the HERV-K Env cytoplasmic tail to acquire oncogenic properties (Lemaitre et al., 2017). This indicates transforming properties of HERV-K Env may contribute to oncogenesis.

HERV-K is the most broadly studied ERV type associated with cancer, followed by HERV-H and HERV-W/syncytin-1. The HERV-K family was transcriptionally active and involved in tumor cell proliferation *via* different mechanisms. HERV-K activation was associated with cancer hallmarks, such as phenotype transition, stemness, immune evasion, and metastatic properties (Balestrieri et al., 2018). Specifically, Rec and Np9 proteins encoded by HERV-K are essential for the control of HERV-K-related cancer stemness features (Balestrieri et al., 2018). Cancer stem cells are required for cancer progression and aggressiveness and express high levels of the stem cell markers, such as *Sox2, Oct4, Nanog*, and kruppel-like factor 4 (Barbato et al., 2019). Interestingly, *Oct4* could in return transactivate HERV-K LTRs and synergistically facilitate HERVK expression (Grow et al., 2015). Argaw-Denboba et al. (2017) demonstrated that HERV-K activation promoted phenotype-switching of melanoma cells and was strictly required to maintain the stemness features of CD133^+^ melanoma cells in response to microenvironmental changes. Matteucci et al. (2018) further confirmed that HERV-K activation was required for maintenance and expansion of a CD133^+^ melanoma cell subpopulation. Dai et al. (2018) demonstrated that the HERV-K single-spliced product Env and double-spliced product Np9 played a major role in angiogenesis and tumorigenesis of Kaposi’s sarcoma. HERV-H has been proven to be an important determinant of the pluripotency of human embryonic stem cells, as well as the reprogramming process of iPSCs (Ohnuki et al., 2014).

HERVs may contribute to the inactivation of tumor suppressor genes or activation of downstream oncogenes by insertional mutagenesis or non-homologous recombination (Ohnuki et al., 2014). Cell-cell fusion was an important source of malignant cell heterogeneity and genetic instability, leading to chromosomal numerical abnormalities, thus involved in cancer progression and metastasis (Bastida-Ruiz et al., 2016). Uncontrolled cell fusion was reported to be stimulated by the fusogenicity of HERV Env in tumors (Grandi and Tramontano, 2018a). Moreover, HERV Env *syncytin-1* can mediate the fusion stage when the extracellular vesicle cargo was delivered into target cells to edit the host gene (Uygur et al., 2019). HERV-K Env was also associated with cellular transformation by inducing the downstream effectors of the MAPK/ERK1/2 pathway, including transcription factors ETV4, ETV5, and EGR1 (Lemaitre et al., 2017).

In some scenarios, HERVs up-regulate immune signals and trigger a subsequent anti-tumor immune response by the viral mimicry process. The replication intermediates and protein products of HERVs in tumors can serve as intrinsic pathogen-associated molecular factors and activate the immune system to recognize HERVs as exogenous infections, thus stimulating the specific anti-tumor immunity (Alcazer et al., 2020; Vergara Bermejo et al., 2020). However, the transmembrane subunits of Env glycoproteins of HERVs possess immunosuppressive properties and contribute to tumor progression (Grandi and Tramontano, 2018a; Vergara Bermejo et al., 2020). In addition, the immunosuppressive domain of HERV Env may abrogate the anti-oncogenic cytolytic immune response to support tumor progression (Kassiotis and Stoye, 2017). It seems that tumors with higher expression of HERVs are more immunogenic. The innate immune systems produce type I/III interferons when they detect the viral products, such as the Env proteins, leading to an antiviral state (Lazear et al., 2019). Thus, HERVs are targeted as a promising internal strategy to enhance the anti-tumor immune responses by sensitizing tumor cells for immunotherapies. Vitiello et al. (2021) reviewed the role of HERVs activation as a promising molecular predictive marker and immunotherapy target in cancers.

## Transcriptional regulation of host genes by human endogenous retroviruses

The human genome consists of 3 billion base pairs of DNA, while only 1% of them can be translated into human proteins. Notably, 25% of the human promoter regulatory regions have TE-derived sequences (Jordan et al., 2003). HERVs and solitary LTRs were the most common retroviral elements in our genome, with the others being classified as non-LTR retrotransposons (Geis and Goff, 2020). Most HERVs have lost the ability of retrotransposition and insertional mutations, but they regulated the host genes by their viral mRNA or protein products, or their LTR-derived gene regulatory regions (Chen et al., 2019; Geis and Goff, 2020). Aberrant expression of oncogenic genes and oncogenic long non-coding RNAs (lncRNAs) were driven by multiple mechanisms, such as gene translocation, gene amplification, and inappropriate usage of tissue-restricted enhancers or promoters (Babaian and Mager, 2016). Exaptation of LTRs as promoters for other protein-coding genes and lncRNAs is frequently observed. Transcriptional regulation by these ancient viral remnants has been demonstrated to be more dynamic and effective than initially assumed. Recent evidence shows that gene co-option of ERVs provides important effects on the transcriptional regulation of the host genes. Kitao et al. (2021) identified an RNA element (SPRE) overlapping with lineage-specific ERVs. The SPRE-like elements not only induced the expression of viral genes but also enhanced host gene expression (Kitao et al., 2021). Bakoulis et al. (2022) demonstrated that co-opted ERVs transcribed into unstable RNAs and also acted as active enhancers and gene promoters.

Here we summarized recent findings regarding the transcriptional regulation of host genes by HERVs. We also provided an overview of how HERVs fulfill physiological functions and modulate genome-wide host gene expression.

### Exaptation of human endogenous retroviruses-long terminal repeats as regulatory regions for host genes

More than 300,000 regulatory regions contain the same sequences as the remnants of inserted LTRs from retroviral infections in our genome. The two LTRs located at the 5′ and 3′ ends of a HERV insert contain transcription factor binding sites (TFBS) to regulate transcription of the insert and adjacent regions efficiently (Rebollo et al., 2012). Solitary LTRs originating from recombination between the 5′ and 3′ LTRs, also function in transcriptional regulation of adjacent cellular genes. More than 800 LTRs from HERVs and mammalian apparent LTR-retrotransposons (MaLRs), a group of retrotransposon-like elements, drive stage-specific gene expression in mammalian oocytes and developing zygotes by acting as alternative promoters and first exons (Franke et al., 2017). Genome-wide studies have estimated that, far more than anticipated, alternative promoters were used in about 75% of human genes (Takeda et al., 2006). We summarized known examples of LTRs that function as alternative promoters for human oncogenes in Table 2. These examples showed that HERV-LTR exaptation induced a new pattern of gene expression different from the pattern before LTR insertion.

**TABLE 2:** Onco-exaptation of LTR-derived oncogenes expression in cancers.

Gene	Primary result of LTR-driven expression	LTR type	Cancer type
IRF5	Ectopic expression of IRF5 protein	(ERV1) LOR1a	Hodgkin lymphoma (Kreher et al., 2014; Babaian et al., 2016)
ALK	Protein truncation	(ERVL) LTR16B2	Melanoma (Wiesner et al., 2015)
CSF1R	Ectopic expression of CSF1R protein	(ERVL-MaLR) THE1B	Hodgkin lymphoma (Lamprecht et al., 2010), melanoma (Giricz et al., 2018)
SLCO1B3/OATP1B3	Cancer-specific expression of a chimeric protein “cancer-type OATP1B3 (Ct-OATP1B3)”	(ERV1) LTR7	Colon and lung cancer tissues (Nagai et al., 2012), colon and pancreatic cancer (Thakkar et al., 2013)
RNF19	Ectopic expression of RNF19 protein	MaLR (LTR) and AluJo elements	Conlon cancer (Huh et al., 2008)
GSDML	Ectopic expression of GSDML protein	HERV-H LTR	Conlon cancer and melanocyte (Sin et al., 2006)
FABP7	Ectopic expression of a chimeric protein “LTR2-FABP7”	(ERV1) LTR2	Diffuse large B-cell lymphoma,(Lock et al., 2014)
Syncytin-1	Overexpression of syncytin-1 through interacting with c-Myb	HERV-W LTR	Bladder urothelial cell carcinoma (Yu et al., 2014)
PLA2G4A	Ectopic Expression of cytosolic phospholipase A2 (cPLA2)	HERV-E LTR	Urothelial carcinoma (Gosenca et al., 2012)
CALB	Ectopic Expression of an aberrant calbindin protein	HERV-H LTR	Prostate carcinoma (Gebefügi et al., 2009)
PLA2L	Ectopic Expression of RTVL-H/PLA2L	RTVL-H LTR	Teratocarcinoma (Feuchter-Murthy et al., 1993)
PTN	Ectopic expression of HERV-PTN chimeric transcripts	HERV type C	Choriocarcinoma (Schulte et al., 1996)
ERRB4	Aberrant expression of ERBB4-truncated transcripts	(ERVL-MaLR) MLT1C LTR	Anaplastic large-cell lymphoma (Scarfo et al., 2016)

Huh et al. (2008) identified a novel alternative *RNF19* promoter region for a MaLR element, and tumor tissues showed a higher expression of the MaLR-derived *RNF19* transcripts compared with normal and primate tissues. In Hodgkin lymphoma, upregulation of the oncogenic factor interferon regulatory factor 5 (*IRF5*) was driven by the transcriptional activation of a normally dormant HERV LOR1a-LTR upstream of *IRF5* (Babaian et al., 2016). Ectopic expression of the solute carrier organic anion transporter family member 1B3 (*OATP1B3*) was detected in solid tumors of non-hepatic origin, particularly in pancreatic cancer and colon cancer (Lee et al., 2008; Thakkar et al., 2013). The expression of “cancer-type” *OATP1B3* was driven by an alternative promoter within the 5′ LTR (LTR7) of a partly full-length antisense HERV-H element (Nagai et al., 2012). Overexpression of fatty acid binding protein 7 (*FABP7*) was detected in several solid tumors (Thumser et al., 2014). *FABP7* is expressed driven by an antisense 5′LTR of a HERV-E element in some diffuse large B-cell lymphoma patients (Lock et al., 2014).

Cancer progression was accompanied by repression of tumor suppressor genes and abnormal expression of oncogenes. Beyer et al. (2016) and Kronung et al. (2016) identified the LTR12 from *ERV9* as a germline-specific promoter that induced the expression of the tumor suppressor gene *TP63* and *TNFRSF10B*, which is often silenced in testicular carcinoma. The promoter activity of LTR12 can be reactivated by broad-range histone deacetylase (HDAC) inhibitors in testicular cancer cells, which represent a novel applicable way to induce activation of pro-apoptotic genes in cancer cells. We also identified an ERV-related human-specific gene, named *psiTPTE22-HERV*, which is silenced by DNA methylation in cancers (Liang et al., 2010).

### Human endogenous retroviruses-derived long non-coding RNAs with oncogenic functions

LTRs regulated the expression of neighbor genes and generated RNAs required for LTR enhancer activity by acting as promoters and enhancers. Song et al. (2002) transfected human tumor cells by a retroviral vector containing *VL30-1* lncRNA, which was transcribed from one member of the VL30 ERV family, *VL30-1* lncRNA was capsulized and integrated into the host genome as an ERV, thereby increasing the metastatic potential of the host. The undifferentiated state for cell identity and pluripotency was maintained by the stem-cell-specific transcripts driven by HERV-LTR promoter-enhancer activity (Pontis et al., 2019). Babarinde et al. (2021) showed that 65% of non-coding transcripts in human pluripotent stem cells (hPSCs) contained TE-derived sequences, and single-cell RNA-seq revealed that hPSCs expressed ERV-containing transcripts, and differentiated subpopulations lacking ERV-containing transcripts. ESRG is an hPSC-related HERV-H-driven lncRNA. It has a higher expression than other HERV-Hs and is tightly silenced after differentiation (Takahashi et al., 2021). Linc-ROR is a lncRNA derived from the LTR7 of a HERV-H element. It was shown to play a role in human pluripotency by acting as a microRNA sponge of miR-145, which inhibits the pluripotency transcription factors *Oct4, Sox2*, and *Nanog* (Wang et al., 2013). Recently, several studies have reported an oncogenic role for lncROR in breast and gastric cancers by various mechanisms (Fan et al., 2015; Zhou et al., 2016). We summarized known examples of ERV-driven lncRNAs that promote tumor progression and those with expression correlated with cancer in Table 3.

**TABLE 3:** HERV-derived lncRNAs with oncogenic functions.

lncRNA	Primary result of lncRNA expression	ERV type	Cancer type
TROJAN	Binds to a metastasis-repressing factor ZMYND8, and increases its degradation.	LTR70 mosaic with MER67C and LTR56	Human triple-negative breast cancer (Jin et al., 2019)
UCA1	Regulates cell cycle by CREB through PI3-K dependent pathway.	LTR7Y and HERV-H	Bladder carcinoma (Yang et al., 2012)
linc-ROR	Induces an epithelial-to-mesenchymal transition (EMT) program and also play a role in human pluripotency.	HERV-H, LTR7	Breast cancer (Hou et al., 2014), gastric cancer (Yu et al., 2020)
lncMER52A	Regulates EMT pathway *via* post-translational control of p120-catenin protein stability.	MER52A LTR	Hepatocellular carcinoma (Wu et al., 2020)
POU5F1-PSORS1C3	Acts as promoter initiating long RNA transcripts through the PSORS1C3-POU5F1.	ERV-LTR2B	Renal cell carcinoma (Siebenthall et al., 2019)
SchLAP1	Inhibits the function of the tumor suppressor SWI/SNF complex.	LTR12C (ERV9)	Prostate cancer (Prensner et al., 2014)
HOST2	Functions as a miRNA sponge of miRNA let-7b, which is a tumor suppressor gene.	HERV-E, LTR2B	Ovarian cancer (Gao et al., 2015)
HERV-H4p15.2	Down-regulated expression in colon, stomach, and kidney cancers.	HERV-H	Colon, stomach, and kidney cancers (Liang et al., 2009b)
HCP5	Enriched in lung cancer risk-related loci (6p21 and 15q25) by GWAS.	ERV type 16	Lung cancer (Yuan et al., 2016)

HERVs are known to harbor cis-regulatory elements, and their roles in modulating innate and adaptive host immunity have been studied recently. HERVs-induced cellular immune responses could result in beneficial or pathogenic effects. “Traditional” genes are co-opted for new uses as they are descendants of ancient retroviral *gag*, *pol*, or *env* genes (Grandi and Tramontano, 2018b). For example, the integrase domains and RNaseH of the retroviral pol gene serve as fundamental blocks of our immune system (Moelling and Broecker, 2015). A new isoform of Angiotensin-converting enzyme 2 (ACE2) was generated by the co-option of intronic retroelements as a promoter and alternative exon, inducing abnormal expression patterns of the aerodigestive tracts and being more sensitive to IFN stimulation (Ng et al., 2020). HIV-1 infection activated several members of the HERV9 lineage, particularly LTR12C elements (Srinivasachar Badarinarayan et al., 2020). *LTR12C* elements provide cryptic transcription start sites for the interferon-inducible genes *GBP2* and *GBP5* in primary CD4^+^ T cells (Srinivasachar Badarinarayan et al., 2020).

### Modulation of genome-wide host gene expression by human endogenous retroviruses

Genome evolution results in the acquisition of new genes or new gene isoforms and new gene expression patterns. TEs contribute to the source of genetic innovation. TEs have their own promoters and enhancers, which can act as “controlling elements” for host genes, as well as their own open reading frames. HERV is a subtype of TEs and also an important source of incoming genetic materials for the host to repurpose, which has co-evolved with host genes for millions of years. Cryptic regulatory elements within HERVs were reactivated in cancers by epigenetic regulations to influence oncogenesis, the process of which is termed as onco-exaptation (Babaian and Mager, 2016). Since epigenetic variance increases during oncogenesis, the epigenetic evolution model for onco-exaptation takes an important place in tumor evolution (Mazor et al., 2016). Babaian et al. (2016) re-analyzed the CAGE datasets of retrotransposon-derived transcriptional start sites (TSSs) published by Faulkner et al. (2009), and demonstrated that TE-derived TSSs had lower expression and were less reproducible between biological replicates, as compared to non-TE promoters (Babaian and Mager, 2016). During malignant transformation, deregulation of transcription factors and genome-wide epigenetic variations were observed (Hanahan and Weinberg, 2011), changing the set of active LTRs and increasing the total level of LTR-driven transcriptional elements. Therefore, LTR-driven transcription would be also subject to epigenetic reprogramming and become “passenger” expression signals during the somatic evolution of cancer cells (Lee and Kong, 2016).

HERVs enabled coordinated genome-wide activation of species-specific gene expressions by providing binding sites for transcriptional regulators. However, the HERV-induced gene expression and regulation must be balanced with their genotoxic potential. Jang et al. (2019) characterized the global profile of TE onco-exaptation across different cancer types and highlighted the TE cryptic promoter-activation events as an important mechanism for oncogene activation and tumorigenesis, including the *HERVH-SLCO1B3* transcript, which was onco-exaptation in various cancer types. Pontis et al. (2019) showed that Krüppel-associated box (KRAB)-containing zinc finger proteins (KZFPs) controlled the activation of transcriptional cis regulators derived from HERV-K and HERV-H subgroups during early embryogenesis. Thus, the transcriptional impacts of HERV-TEs during embryogenesis facilitated their incorporation into the genome-wide transcriptional networks, thus regulating the human genome.

## Reverse transcription and retrocopying by reverse transcriptase of human endogenous retroviruses

Mammal genomes have an ancient history of co-evolution with ERVs, resulting in ERVs comprising a substantial fraction of most mammal genomes (Bourgeois et al., 2020). Thus, interest has been intrigued in exploring mechanisms about how the host genomes keep pace with the rapidly evolving viruses. The life cycle of HERVs contains transcription by RNA polymerase II, RT, and re-integration (Weiss, 2017). RT is the defining activity of retroviruses. Viral genomic RNAs are converted into double-stranded proviral DNAs intermediated by this process. The provirus is required for virus replication, permanent integration into the host cell chromosome, and further expression by the host cell transcriptional machinery (Baltimore, 1970). The retroviruses can trigger tumorigenesis by inserting their proviral DNA into host genomic regions (retroviral integration) that control the expression of proto-oncogenes (Lesbats et al., 2016). Thus, there remains a pressing need for a deeper molecular understanding of the HERV life cycle. Here we focus on recent advances in understanding how HERV RT initiates, the roles of HERV RTase and the host tRNA-derived fragments (tRFs) in this process, and the functions of RT and retrocopying by HERVs.

### Reverse transcription of human endogenous retroviruses mediated by their own reverse transcriptase and primed by host tRNAs

RTase is abundantly distributed in organisms that are with high copy numbers of mammalian retroelements (Lander, 2011). Activities of endogenous RTase encoded by retrotransposons have been identified in the cells of higher eukaryotes and associated with a wide spectrum of pathological and physiological processes (Spadafora, 2008). RTase enzymes have evolved to bind specific host tRNAs with high affinity to initiate RT. Primer tRNAs are enriched in viral particles with RTase in cells infected with exogenous retroviruses, such as HIV, respiratory syncytial virus, and human cytomegalovirus (Kelly et al., 2003). HERVs shape mammalian genomes in a random way as the integration of exogenous retroviruses and modifications of endogenous retroviruses may both occur during the evolution of the host genome. Co-option of HERVs seems to occur in a gradual process, and they may achieve cis-regulatory effects immediately after integration into the host genome or acquire the regulatory capability until additional mutations occur (Chuong et al., 2017). Goke et al. (2015) reported that ERVs were systematically transcribed during human early embryogenesis in a stage-specific manner. The expression of specific ERVs was identified as a mark of cellular identity and cell potency that characterized the cell populations in early embryos and placentation in mammalians (Lynch et al., 2015). These lineage-specific co-opted HERVs are integrated into the host genome under similar selective pressures (Chuong et al., 2017). ERVs, like exogenous infectious retroviruses, use their host tRNAs as primers for RT and replication. ERVs initiate RT by using their specific tRNA primers and copying their RNA into DNA to insert into the genome (Schorn and Martienssen, 2018).

**FIGURE 2:**
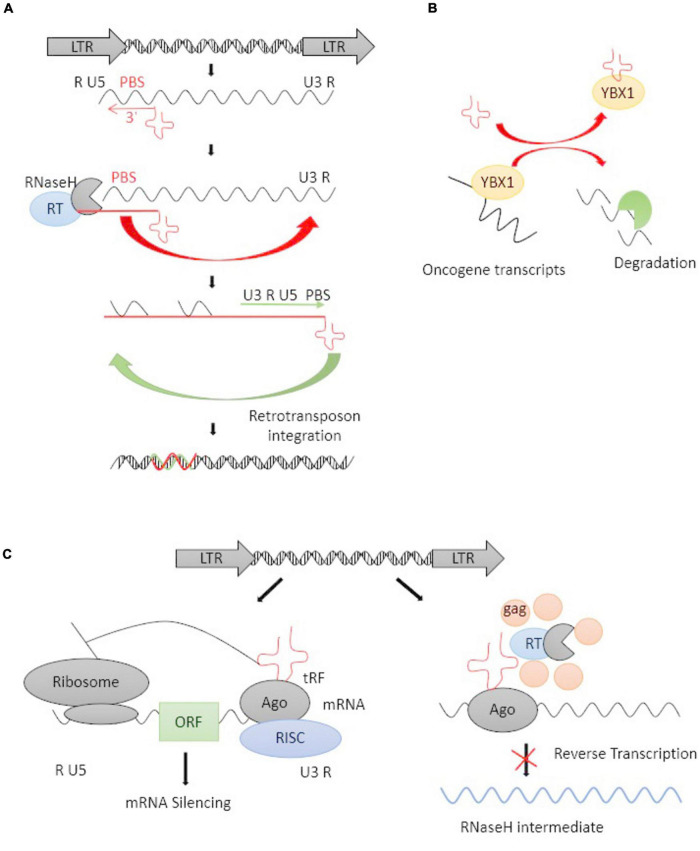
The life cycle of an LTR retrotransposon and tRFs-mediated inhibition. **(A)** Schematic representation of the life cycle of an LTR retrotransposon. The LTR encodes promoter elements and termination signals. The 3′-tRFs (red cloverleaf) primes RT by hybridizing to PBS. After transfer events, a double-stranded retroviral DNA is integrated into the host genome. **(B)** tRFs competitively bind with YBX1 and caused oncogene transcripts degradation. **(C)** tRFs function as potential inhibitions at the posttranscriptional and RT levels. tRFs loaded into argonaute (AGO) protein and other proteins form an RNA-induced silencing complex (RISC), thus inducing RNAi on target mRNAs (right panel). tRFs inhibit RT by directly targeting their PBS and inhibiting retrotransposon’s replicative cycle (left panel).

tRFs, a novel type of mature tRNA-derived or precursor tRNA-derived small non-coding RNAs, play an important role in governing gene expression at a post-transcriptional level (Xie et al., 2020). The 3′-end, but not 5′-end, of mature tRFs are highly complementary to HERV sequences in the genome. 3′-tRFs perfectly match ERV-LTRs at their highly conserved tRNA PBS (Schorn and Martienssen, 2018), which plays an important role in RT. HERV-derived sequences bind specific host tRNAs with high affinity and recruit them to the PBS site, where the 3′-tRFs initiate RT and integrate the full-length, double-stranded retroviral DNA into the genome (Figure 2A; Lesbats et al., 2016; Schorn et al., 2017; Schorn and Martienssen, 2018). 3′-tRFs have been found in stem cells and cancer cells with a high HERV burden (Schorn et al., 2017). Schorn et al. (2017) reported that 3′-tRFs protected the pre-implantation embryo from transposon damage during epigenetic reprogramming. It is known that changes in tRNA levels can promote cancer (Goodarzi et al., 2016), and cleavage into tRFs is expressed highly in a wide range of cancer cell lines, playing important roles in tumorigenesis by regulating the expression of oncogenes (Zhu et al., 2019).

### Inhibition of reverse transcription of human endogenous retroviruses *via* host tRNA-derived fragments

tRFs targeting is a highly conserved mechanism of small RNA-mediated transposon control, PBS of ERVs may offer a unique target for specific inhibition of ERV RT and retrotransposon mobility (Yang and Kazazian, 2006; Schorn et al., 2017). This reminds us of the possibility that replication-competent ERV sequences with a functional PBS can be blocked at the RT step by targeting the PBS sequence. Schorn et al. (2017) showed that tRFs targeted various ERVs, particularly active ERVs which can cause ongoing mutagenesis, and inhibit retrotransposition by obstructing RT.

Recently, tRFs are used for the promotion and suppression of retrotransposon transcription (Schorn et al., 2017). Li et al. (2012) demonstrated that endogenous 3′-tRFs suppressed the unwarranted expression of ERVs through the RNA interference (RNAi) pathway, thus protecting the genome against retrotransposons. There are two different classes of 3′-tRFs: 17–19 nt and 22 nt fragments, carrying 1-methyladenosine and pseudouridine at positions of 17–19 and 22, respectively (Mboko et al., 2014; Kumar et al., 2016). Endogenous 22-nt 3′-tRFs post-transcriptionally silence coding-competent ERVs, while 18 nt 3′-tRFs have no effect on RNA or protein levels but specifically interfere with RT (Schorn et al., 2017). Therefore, three possible ways have been proposed for tRFs-inhibited retrotransposition: (1) tRFs-guided H3K9me3 deposition in transcriptional silencing, (2) RNAi-induced post-transcriptional silencing, (3) Blocking RT to inhibit the retroviral intermediates.

Krishna et al. (2021) revealed the involvement of functional tRFs in transposon post-transcriptional control. Goodarzi et al. (2015) found a specific set of tRFs functionally combined with the oncogenic RNA-binding protein YBX1, which was one of the most highly expressed oncogenes in human cancer, it can bind with some endogenous oncogene transcripts to maintains their stability and promote cancer progression (Lasham et al., 2012). tRFs can competitively bind with YBX1 and caused oncogene transcripts degradation (Figure 2B). Argonaute (AGO) proteins are loaded with small RNAs to silence complementary RNA transcripts, and they are central to RNAi. The dysregulated expression of the genes encoding AGO proteins was demonstrated in solid tumors as well as leukemia (Nowak and Sarshad, 2021). The majority of reports agree that 3′-tRFs could be incorporated into AGO proteins and act through RNAi pathways (Martinez et al., 2017). Cross-linking ligation and sequencing of hybrids (CLASH) and high-throughput RNA sequencing have revealed that tRFs target a number of genic mRNAs in humans (Kumar et al., 2014). Some reports revealed that tRFs may have a parallel effect on inhibiting translation in a miRNA-like fashion and inducing mRNA cleavage (Figure 2C; Li et al., 2012, 2013). tRFs are also incorporated into AGO proteins and target retrotransposons by inducing the production of secondary sRNAs from their RNA transcripts. A lack of mature tRNAs can inhibit the translation of retrotransposon proteins and prevent RT at the same time (Figure 2C). Thus, the ability of 3′-tRFs, *via* degrading ERV mRNAs, to inhibit RT and retrotransposon mobility could be a highly conserved mechanism for controlling the transposons process.

### Reverse transcriptase inhibitors in inhibiting endogenous retrovirus activity

Antiviral compounds or RTase inhibitors inhibit endogenous RTase by various mechanisms. Interferon regulatory factor-1 (*IRF-1*) is an antiviral host factor that attenuates the replication of multiple RNA and DNA viruses and acts as a tumor suppressor (Wang et al., 2007; Mboko et al., 2014). Stoltz et al. (2019) further identified *IRF-1* as a suppressor of ERV expression, which may contribute to its tumor-suppressive function considering the emerging appreciation of the oncogenic role of ERVs. Tyagi et al. (2017) found that RTase inhibitors could significantly inhibit HERV-K RTase activity, while protease inhibitors were not as effective as RTase and integrase inhibitors. The RTase inhibitors and the integrase inhibitor could effectively block pseudotyped HERV-K virus infection and production in HeLa cells, such as Zidovudine, Abacavir, and Raltegravir (Tyagi et al., 2017). The non-nucleoside RTase inhibitor, Efavirenz, alone or in combination with other drugs could reduce the multiple sclerosis-related retrovirus (HERV-W) env expression *in vitro* (Morandi et al., 2018). Morandi et al. (2018) observed that people infected with HIV may have a lower risk of developing multiple sclerosis than the HIV-uninfected healthy population, supporting the hypothesis that anti-retroviral therapies used to treat HIV infection suppress HERV expression as well.

### Reverse transcription of host genes by endogenous retrovirus to mediate retrocopying

HERVs residing in human genomes “copy-and-paste” themselves *via* the activation of RTase. In addition to acting on their RNAs to replicate, HERVs also occasionally act on host mRNAs. RTase of HERVs facilitates the duplication of host genes *via* RT of the mRNA and integration of the cDNA, the process of which is termed retro copying (Casola and Betran, 2017). Retrocopying by HERV RTase can inflict deleterious consequences on host genomes by disrupting genes, causing insertional mutagenesis and ectopic recombination (Hancks and Kazazian, 2012). However, sometimes retrocopying can introduce innovation in host genomes *via* the birth of new exons or genes and gene-regulatory networks (Mi et al., 2000; Schmitz and Brosius, 2011; Chuong et al., 2016). Yang et al. (2020) showed that retrotransposon-mediated gene birth could lead to the continual evolution of new innate immune genes.

Retrocopying is different from DNA-based duplications, with limited RNA expression in germline cells and early embryonic tissues (Friedli et al., 2014). Functional retention in retrocopied sequences has been found. For example, novel *TRIMCyp* fusion genes were created by retrocopying of the *CypA* gene between coding exons of the TRIM5 gene, thus retroviruses including HIV-1 were restricted (Malfavon-Borja et al., 2013). In other examples, ERV elements and viral genes themselves, such as the LINE-1 type transposase domain containing 1 (*L1TD1*) and *Refrex-1* (encoding a truncated envelope protein) genes, have been retrocopied and domesticated for various functions, such as antiviral defense (Ito et al., 2013; McLaughlin et al., 2014).

## Discussion

Most HERVs are silenced due to accumulated mutations, deletions, truncations, and fusions during the evolution to maintain genomic stability or epigenetic mechanisms. HERVs are reactivated in certain pathological contexts, such as cancers or virus infections. Cancer cells are characterized by deregulation of the genome (Rodriguez-Paredes and Esteller, 2011), which changes the gene expression patterns, including HERVs. The balance between HERVs and the genome is broken in cancers. Reactivation of HERVs can be induced by genetic deregulation in cancers, which we have summarized in our previous review (Zhang et al., 2019). Treatment of DNMT- and HDAC-inhibitors can result in HERV expression in somatic cells (Groudine et al., 1981; Daskalakis et al., 2018), which reminds us that HERV expression can also be induced by epigenetic deregulation of the genome. The roles of HERVs reactivation have been partially explored, including (1) transcriptional regulation of host genes, (2) acting as new binding sources of transcription factors, (3) binding specific tRNAs to initiate RT, and (4) facilitating retrocopying of host genes. More details about the mechanisms by which HERVs function in oncogenic progress are still not fully understood, and new HERV-related regulators remain to be identified.

## Author contributions

MZ: conceptualization, funding acquisition, and writing—original draft. MZ and JL: investigation. SZ and JL: supervision. MZ, SZ, and JL: writing—review and editing. All authors contributed to the article and approved the submitted version.

## Conflict of Interest

The authors declare that the research was conducted in the absence of any commercial or financial relationships that could be construed as a potential conflict of interest.

## Publisher’s Note

All claims expressed in this article are solely those of the authors and do not necessarily represent those of their affiliated organizations, or those of the publisher, the editors and the reviewers. Any product that may be evaluated in this article, or claim that may be made by its manufacturer, is not guaranteed or endorsed by the publisher.
